# Hyaluronan-Based Gel Promotes Human Dental Pulp Stem Cells Bone Differentiation by Activating YAP/TAZ Pathway

**DOI:** 10.3390/cells10112899

**Published:** 2021-10-26

**Authors:** Marcella La Noce, Antonietta Stellavato, Valentina Vassallo, Marcella Cammarota, Luigi Laino, Vincenzo Desiderio, Vitale Del Vecchio, Giovanni Francesco Nicoletti, Virginia Tirino, Gianpaolo Papaccio, Chiara Schiraldi, Giuseppe Andrea Ferraro

**Affiliations:** 1 Department of Experimental Medicine, Section of Biotechnology, Molecular Medicine and Medical Histology, University of Campania “L. Vanvitelli”, Via L. de Crecchio 7, 80138 Naples, Italy; marcella.lanoce@unicampania.it (M.L.N.); antonietta.stellavato@unicampania.it (A.S.); valentina.vassallo92@gmail.com (V.V.); marcella.cammarota@unicampania.it (M.C.); vincenzo.desiderio@unicampania.it (V.D.); vitale_84@hotmail.it (V.D.V.); chiara.schiraldi@unicampania.it (C.S.); 2 Multidisciplinary Department of Medical-Surgical and Dental Specialties, University of Campania “L. Vanvitelli”, Via L. de Crecchio 6, 80138 Naples, Italy; luigi.laino@unicampania.it (L.L.); giovannifrancesco.nicoletti@unicampania.it (G.F.N.); giuseppe.ferraro@unicampania.it (G.A.F.)

**Keywords:** hyaluronic acid, dental pulp stem cells, osteogenic differentiation, YAP/TAZ pathway

## Abstract

Background: Hyaluronans exist in different forms, accordingly with molecular weight and degree of crosslinking. Here, we tested the capability to induce osteogenic differentiation in hDPSCs (human dental pulp stem cells) of three hyaluronans forms: linear pharmaceutical-grade hyaluronans at high and (HHA) low molecular weight (LHA) and hybrid cooperative complexes (HCC), containing both sizes. Methods: hDPSCs were treated with HHA, LHA, HCC for 7, 14 and 21 days. The effects of hyaluronans on osteogenic differentiation were evaluated by qRT-PCR and WB of osteogenic markers and by Alizarin Red S staining. To identify the involved pathway, CD44 was analyzed by immunofluorescence, and YAP/TAZ expression was measured by qRT-PCR. Moreover, YAP/TAZ inhibitor-1 was used, and the loss of function of YAP/TAZ was evaluated by qRT-PCR, WB and immunofluorescence. Results: We showed that all hyaluronans improves osteogenesis. Among these, HCC is the main inducer of osteogenesis, along with overexpression of bone related markers and upregulating CD44. We also found that this biological process is subordinate to the activation of YAP/TAZ pathway. Conclusions: We found that HA’s molecular weight can have a relevant impact on HA performance for bone regeneration, and we unveil a new molecular mechanism by which HA acts on stem cells.

## 1. Introduction

Hyaluronic acid (HA) is a key component of extracellular matrix (ECM) in the majority of human tissues [1].

It interacts with other macromolecules and plays a predominant role in tissue morphogenesis, cell migration, differentiation, and adhesion [2].

HA structure consists of repeated disaccharide unit of *N*-acetyl-glucosamine and glucuronic acid, linked through a betaglycosidc bond [3].

However, what we define as hyaluronic acid is not a single and unique molecule with distinct characteristics, but rather a family of macromolecules with different molecular weight and biological activity [4]. HA’s molecular weight may substantially affect its biochemical features and its effects on biological processes and tissue regeneration.

The difference in linear hyaluronan polymers is related to the extent of repeated units, thus high molecular weight hyaluronan (HHA) refers to biopolymers of 1 MDa or above, while low molecular weight hyaluronan (LHA) generally refers to 100 KDa average molecular weight molecules. Hybrid cooperative complexes of hyaluronic acid (HCC) were obtained through a patented, thermal procedure for the formation of stabilized cooperative complexes (hydrogen bond formation between the two diverse HA chains) of hyaluronic acid, starting from an initial mixture of an equal amount of HHA and LHA [5].

HA-based products have already reached clinical-grade approval and are used in many fields such as orthopedics, ophthalmology, dermatology, and plastic surgery [6]. However, most of these products do not report HA’s molecular weight or report a generic reference to it, such as high, medium and low without a general consensus on what this term means. Moreover, different products with different HA molecular weights are approved for the same indication, which, in light of recent knowledge, makes little sense. For these reasons, a clarification on the biological properties of different molecular weights in HA is of greatest importance to improve existing products and formulate new and more effective ones. To give an example, different formulations of HA, with diverse molecule weights, stimulate adipose stem cells (ASCs) to differentiate in adipose tissue [7], or preserve the stemness of human mesenchymal stem cells (hMSCs) [8].

hMSCs have attracted attention in tissue regeneration for their proliferation potential and the ability to differentiate in several cytotypes such as osteoblasts, adipocytes, chondrocytes, and endothelial cells [9]. Among hMSCs, human dental pulp stem cells (hDPSCs) were shown to be remarkably suitable for bone-endothelium co-differentiation [10]. hDPSCs can be easily extracted from dental pulp, and have several advantages compared to other types of hMSCs: better proliferative potential [11,12], a simpler primary isolation method [13], and a higher success rate in long-term in vitro culture. Moreover, hDPSCs can be stored for a long time without losing their stemness [14].

YAP/TAZ (yes-associated protein/transcriptional coactivator with PDZ binding motif) pathway is involved in transmitting extracellular mechanical signals to the nucleus to regulate different biochemical signals, among which osteogenic differentiation [15]. They interact with transcription factors, including transcription enhancer factors/transcriptional enhanced associate domain, runt domain transcription factors (RUNX), and peroxisome proliferator-activated receptors γ (PPAR γ), regulating downstream gene expression [16]. Different studies suggested that YAP/TAZ activation could be related to cell shape, matrix stiffness, and mechanical stimulation, regulating cell proliferation, differentiation, and apoptosis [17,18].

Here, we evaluated the biological activity of hybrid cooperative complexes (HCC) gels, in comparison to high (HHA) and low (LHA) HA molecular weight on hDPSCs.

We found that HCC improves osteoblastic differentiation of hDPSCs via activation of the YAP/TAZ pathway. Our findings highlight the importance of HA’s molecular weight and unveil a new molecular mechanism underlying the biological effect of HA on hDPSCs osteogenic differentiation. This opens a new scenario for clinical applications of HA-based gels in regenerative medicine.

## 2. Materials and Methods

### 2.1. Reagents

High and low molecular weight hyaluronic acid (HA) were provided by Altergon (Altergon s.r.l., Avellino, Italy). These are fermentative HA of high purity derived from *Streptococcus equi* ssp. *equi*, extensively purified at pharmaceutical grade (e.g., purity > 95%, water content < 10%, EU/mg < 0.05, and low metal contents). The raw materials were fully characterized through hydrodynamic analyses using Size-exclusion chromatography coupled to a triple detector (SEC-TDA, Viscotek Malvern, Malvern, UK) [19]. Hybrid cooperative complexes of hyaluronic acid (HCC), obtained through a thermal procedure for the formation of hybrid cooperative complexes of hyaluronic acid, starting from an initial mixture of an equal amount (ratio 1:1) of HHA (Mw = 1400 ± 200 kDa; Mw/Mn = 1.4) and LHA (Mw = 100 ± 20 kDa; Mw/Mn = 1.4). The starting concentration used was 32 g/L: 16 mg HHA + 16 mg LHA in 1 mL volume. The final concentration used in these experiments was 1.6 mg/mL obtained by opportunely diluting all solutions with the culture medium (DMEM Dulbecco’s modified Eagle’s medium, Gibco, Invitrogen, and/or OM osteogenic medium).

### 2.2. Dental Pulp Extraction and Culture

All experimental procedures involved were approved by the Ethics Committee of University of Campania approved on 12 June 2005, Internal Registry: Experimentation #914 and were performed in line with the principles of the Declaration of Helsinki.

Human dental pulps were extracted from teeth of healthy adults (aged 21–38 years) as described previously [13]. All participants signed the Ethical Committee (University of Campania Internal Ethical Committee) consent form. Every participant was pretreated for a week with professional dental hygiene. The dental crown was covered with 0.3% chlorhexidine gel (Forhans) for 2 min before the extraction. Dental pulp was obtained with a dentinal excavator or a Gracey curette. The pulp was delicately removed and immersed for 1 h at 37 °C in a digestive solution composed of 3 mg/mL of type I collagenase and 4 mg/mL of dispase in PBS containing 40 mg/mL of gentamicin. Once digested, the solution was filtered through 70 μm Falcon strainers (Becton & Dickinson). Cells were cultured in basal growth medium (standard medium) consisting of Dulbecco’s modified Eagle’s medium (DMEM) with 100 units/mL of penicillin, 100 mg/mL of streptomycin, and 200 mM l-glutamine (all from Gibco, Rodano, Milan, Italy), supplemented with 10% heat-inactivated AB-HS (Invitrogen). Cells were maintained in a humidified atmosphere under 5% CO_2_ at 37 °C, and the media were changed twice a week.

### 2.3. hDPSCs Isolation and Osteogenic Differentiation

At the first passage of culture, cells were detached using trypsin–EDTA (GIBCO). At least 200,000 cells were incubated with fluorescent-conjugated antibodies for 30 min at 4 °C, washed, and resuspended in PBS. The antibodies used in this study were: anti-CD34 PE (BD Pharmingen, Buccinasco, Milano, Italy, 555822), anti-CD90 FITC (BD Pharmingen, Buccinasco, Milano, Italy, 555595) and anti-CD45 APC-Cy7 (BD Pharmingen, Buccinasco, Milano, Italy, 557833). Isotypes were used as controls. Cells were analyzed with FACS ARIA III (BD Biosciences, San Jose, CA, USA) and data collected with Diva Software. Cells were sorted using simultaneous positivity for CD90 and CD34 using a FACS ARIA III (BD, Franklin Lakes, NJ, USA) as previously reported [13]. The purity of sorted populations was routinely 90%.

hDPSCs were treated with HHA, LHA, HCC diluted in DMEM and osteogenic media (OM). All experiments were performed at 7, 14 and 21 days. Media were changed twice a week. Cells grown in DMEM or OM without hyaluronans were used as controls.

For osteogenic differentiation, hDPSCs were cultured in osteogenic medium containing 100 nmol/L dexamethasone, 10 mmol/L beta-glycerophosphate, and 0.05 mmol/L L-ascorbic acid-2-phosphate. Osteogenic differentiation was evaluated by Alizarin Red S (ARS) and the expression of bone-related markers such as osteocalcin (OC), osteopontin (OPN), and bone sialoprotein (BSP) at 7, 14 and 21 days. For Alizarin Red S staining, samples were washed twice in PBS, fixed with 4% paraformaldehyde (PFA) for 30 min at 4 °C, and stained with 2% Alizarin Red solution, pH 4.2 (Sigma Aldrich, Milan, Italy) for 20 min at room temperature. Stained cells were extensively washed with deionized water to remove any non-specific precipitation. Micrographs were taken using a microscope Eclipse TE2000-S (Nikon) and a Nikon camera. The number of bone nodules and area calculation of positive staining were measured and compared amongst groups. Quantification of the staining intensity was measured using ImageJ software (National Institutes of Health (NIH), Bethesda, MD, USA).

### 2.4. YAP/TAZ Inhibitor-1 Treatment and Cell Viability Assay

To confirm involvement of YAP/TAZ pathway in promoting osteogenic differentiation, hDPSCs were treated with hyaluronans and YAP/TAZ inhibitor-1 (MedChemExpress, Segrate, Milan, Italy.). To evaluate the cytoxicity of YAP/TAZ inhibitor-1, hDPSCs viability was measured by the colorimetric 3-(4,5-dimethyl-2-thiazolyl)-2,5-diphenyltetrazolium bromide (MTT) assay. Cells were seeded in 96-well plates at a density of 10^4^ cells per well, treated with different concentrations (50, 25, 12.5, 7.25, 5, 2.5, 1.25, 0.75 nM) for 24, 48, 72 h and 7 days. After incubation, they were treated with 100 μL of 1 mg/mL MTT (Sigma) in DMEM medium containing 10% FBS for 4 h at 37 °C. The medium was then replaced with 200 μL of DMSO and shaken for 15 min. Absorbance at 540 nm was measured using a microplate ELISA reader with DMSO used as the blank.

YAP/TAZ inhibitor-1 treated hDPSCs were cultured in DMEM at 10% FBS for 24, 72 h and 7 days. *YAP, TAZ, RUNX-2, OC, OPN*, and *BSP* gene expression were evaluated by real Time PCR. OC, OPN, and BSP protein levels were checked also by western blotting.

### 2.5. RNA Extraction and Quantitative Real-Time PCR Analyses

hDPSCs treated with HHA, LHA, and HCC (0.16% *w*/*v*) for 7, 14, and 21 days, and with YAP/TAZ inhibitor-1 for 24, 72 h and 7 days, were directly lysed with TRIzol^®^ (Invitrogen, Milan, Italy). Following precipitation with isopropyl alcohol and washing with 75% ethanol, the RNA pellets were re-suspended in nuclease-free water. The concentration of the extracted RNA was determined through a Nanodrop spectrophotometer (Celbio, Milan, Italy) and 1 µg of DNase-digested total RNA was retro-transcripted in the cDNA using Reverse Transcription System Kit (Promega, Milan, Italy). Quantitative real-time PCR was obtained by iQTM SYBR^®^ Green Supermix (BioRad Laboratories Srl, Milan, Italy) to analyze the gene expression of some osteogenic biomarkers such as Osteocalcin (*OC)*, Osteopontin (*OPN*) and Bone sialoprotein (*BSP*). In addition, yes-associated protein (*YAP*) and transcriptional co-activator with PDZ-binding motif (*TAZ*), were analyzed together with the two YAP/TAZ regulated genes, namely the connective tissue growth factor (*CTGF*) and ankyrin repeat domain-containing protein 1 (*ANKRD-1*). Finally, runt domain transcription factors (*RUNX-1* and 2) were evaluated.

Oligonucleotide sequences used for quantitative PCR are reported in Table 1 and were obtained by Beacon DesignerTM software (BioRad Laboratories Srl, Milan, Italy). 

Samples were analyzed in triplicate and the expression of specific mRNA relative to the control was determined after normalization with *HPRT* housekeeping gene (internal control). The fold-change of mRNA expression of the genes under evaluation was calculated by using the 2^−∆∆Ct^ comparative threshold method (∆Ct = difference of ∆Ct between treated cells and untreated cells used as controls). The results were expressed as normalized fold expression, calculated by the ratio of crossing points of amplification curves of several genes and internal standard, by using the Bio-Rad iQTM5 software (Bio-Rad Laboratories Srl, Milan, Italy) as previously reported [7].

### 2.6. Protein Levels Evaluation of OC, OPN, and BSP by Western Blotting Analyses

Following HA-based gels and YAP/TAZ inhibitor-1 treatments, hDPSCs were lysed by a Radio-Immunoprecipitation Assay (RIPA buffer) (1×) (Cell Signaling Technology), protein concentrations were evaluated using the Bradford method, and the western blotting analyses were performed according to previously described protocols [1]. In particular, 30 μg of intracellular proteins were electrophoretically resolved on 15% SDS-PAGE and transferred to a nitrocellulose membrane (GE, Amersham, UK). This latter was blocked with 5% nonfat milk in Tris-buffered saline and 0.05% Tween-20 (TBST) and primary antibodies to detect OC (Abcam, Cambridge, UK, ab93876), OPN (Abcam, Cambridge, UK, ab214050) and BSP (Sigma-Aldrich, AV36681) were used at 1:500 dilutions and incubated for 2 h at room temperature (RT). The nitrocellulose membrane was extensively washed with TBST and immunoreactive bands were detected by chemiluminescence using corresponding horseradish peroxidase-conjugated secondary antibodies (Santa Cruz Biotechnology, Heidelberg, Germany, sc-2005 mouse, sc-2357 rabbit), diluted 1:10,000 for 1 h, at RT and reacted with an ECL system (Millipore, Milan, Italy). Protein levels were normalized versus the signal obtained with an anti-Actin antibody 1:500 dilutions (Santa Cruz Biotechnology, Heidelberg, Germany, sc-8432) and anti-GAPDH antibody diluted 1:500 (Sigma-Aldrich, G8795). The semi-quantitative analysis of protein levels was carried out using the Gel Doc 2000 UV System according to the manufacturer’s protocol.

### 2.7. CD44 Expression in hDPSCs by Immunofluorescence Staining

Monolayers of hDPSCs were cultured and treated with HA-based gels in standard and osteogenic media in four-well covered glass chamber slides. After 48 h of treatment, the cells were fixed with 4% *w*/*v* paraformaldehyde in phosphate-buffered saline (PBS) for 15 min at room temperature and permeabilized in 0.2% Triton X-100 *v*/*v* in PBS for 1 h. Non-specific sites were blocked using blocking buffer solution (PBS containing 10% *v*/*v* bovine serum and 1% *w*/*v* BSA). The cells were then incubated with anti-CD44 antibody (Cell signaling, Leiden, The Netherlands, 156-3C11) overnight at 4 °C followed by incubation with corresponding secondary antibody for 2 h at room temperature. Nuclei were stained with 20-(4-hydroxyphenyl)-5-(4-methyl-1-piperazinyl)-2.50-bi-1H-benzimidazole trihydrochloride hydrate, bisBenzimide (Hoechst 33342, Milan, Italy) and actin filaments were stained using phalloidin tetramethylrhodamine B isothiocyanate (Sigma-Aldrich, Milan, Italy, 51927). Fluorescence images were captured using a fluorescence microscopy system (Nikon, Tokyo, Japan).

### 2.8. YAP-TAZ Expression in hDPSCs by Immunofluorescence Staining

hDPSCs were cultured with HA-based gels in standard medium and treated with YAP/TAZ inhibitor-1 in 24-well covered glass chamber slides. After 72 h of treatment, the cells were fixed with 4% *w*/*v* paraformaldehyde in phosphate-buffered saline (PBS) for 15 min at room temperature and permeabilized in 0.2% Triton X-100 *v*/*v* in PBS for 1 h. Non-specific sites were blocked using blocking buffer solution (PBS containing 10% *v*/*v* bovine serum and 1% *w*/*v* BSA). The cells were then incubated with anti-YAP (PA5-78321) and anti-TAZ antibodies (703032) (Invitrogen) overnight at 4 °C followed by incubation with corresponding secondary antibody for 2 h at room temperature. Nuclei were counterstained with 20-(4-hydroxyphenyl)-5-(4-methyl-1-piperazinyl)-2.50-bi-1H-benzimidazole trihydrochloride hydrate, bisBenzimide (Hoechst). Cells were imaged with a fluorescence microscope EVOS FL Cell Imaging System (Thermo Scientific, Rockford, IL, USA).

### 2.9. Statistical Analysis

Values are shown as the mean ± SD of measurements of at least three independently performed experiments to avoid possible variation of cell cultures. Student’s *t* test was employed, and *p* < 0.05 was considered to be statistically significant.

## 3. Results

### 3.1. hDPSCs Isolation and Cell Culture

At the first culture passage, cells were characterized by evaluating the expression of CD90, CD34 and CD45 using cytometric analysis. Cells were all positive for the mesenchymal markers CD90 and negative for CD45. The expression of CD34 was approximately 18%. This characterization is of paramount importance because only these cells constitute the stromal–vascular fraction of the dental pulp and are able to differentiate toward bone and vessels [11,13].

All experiments were performed using CD34^+^CD90^+^ hDPSCs at 1st passage of culture.

### 3.2. Hyaluronan-Based Gels Positively Affected the Expression of the Osteogenic Markers

To evaluate the ability of the different HA-based gels to affect osteogenic differentiation, we analyzed OC, OPN, and BSP gene and protein expression at 7, 14, and 21 days.

**Figure 1:**
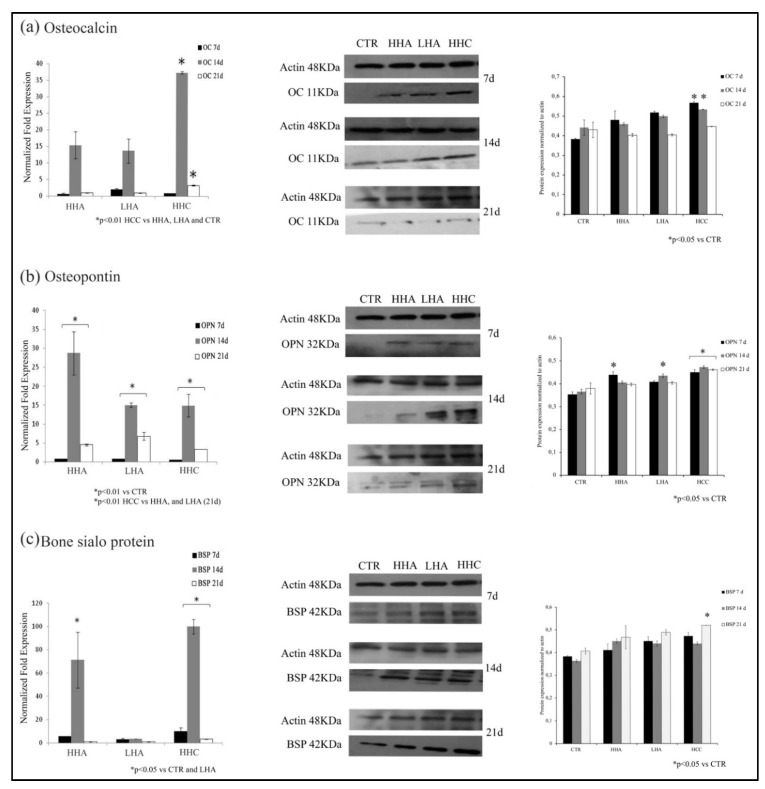
Analysis of *OC*, *OPN* and *BSP* gene expression at 7, 14 and 21 days by qRT-PCR and western blotting in standard medium. (**a**) *OC* gene expression was upregulated with a paramount activity of HCC at 7 and 14 days of treatment both at gene and protein level; (**b**) *OPN* gene and protein expression increased after HA treatment in 14 days; (**c**) *BSP* gene expression increased strongly at 14 days after HHA and HCC treatment, while protein expression is slightly higher in all HA treatments. The results are expressed as the mean ± SD of three independent experiments. * *p* < 0.01 versus CTR and other hyaluronans.

We found that, in standard medium, all hyaluronans prompted an upregulation of *OC* gene expression at 14 days. In particular, HCC induced a significant increase of *OC* gene expression than untreated, HHA and LHA treated cells (*p* < 0.01) at 14 and 21 days (Figure 1a). We next evaluated the protein expression and confirmed qRT-PCR data. Indeed, after 7 and 14 days of treatment, OC levels were slightly increased with HHA and LHA with respect to control, but HCC induced a stronger upregulation with respect to control at 7 and 14 days (Figure 1a).

We next examined OPN expression, another important protein of bone extracellular matrix. We found that, in standard medium, all hyaluronans promoted *OPN* mRNA upregulation at 14 days with a stronger expression induced by HHA (*p* < 0.01). At 21 days only slight differences were evidenced (Figure 1b). Conversely, OPN protein expression was already strongly upregulated with HHA and HCC at 7 days. Then, at 14 days, LHA and HCC promoted its increase, whereas at 21 days, only HCC significantly upregulated OPN expression, compared to control and the other hyaluronans treatments (Figure 1b).

BSP is the main component of bone mineralized matrix. In our models, we found that also *BSP* gene expression was strongly upregulated by HCC after 7 and 14 days of treatment (Figure 1c). Conversely, at 7 and 14 days, BSP protein levels were slightly increased by all hyaluronan-based treatments and after 21 days, only HCC was effective in increasing BSP protein expression (*p* < 0.05) with respect to HHA, LHA, and control (Figure 1c).

In osteogenic medium, there were no strong differences among treatments and controls (Figure S1).

Regarding gene expression evaluation, only slight differences were evident starting from 14 days for all the genes considered. No significant differences were appreciable at protein level.

Taken together, our results show that all hyaluronan-based gels positively modulated the bone-related genes and protein expression in standard medium with a paramount activity of HCC.

### 3.3. Hyaluronan-Based Gels Induced an Increase of Calcification Nodules in hDPSCs

To better characterize the osteogenic differentiation, we evaluated the ability of hDPSCs to form calcification nodules after hyaluronan-based gels treatment.

**Figure 2:**
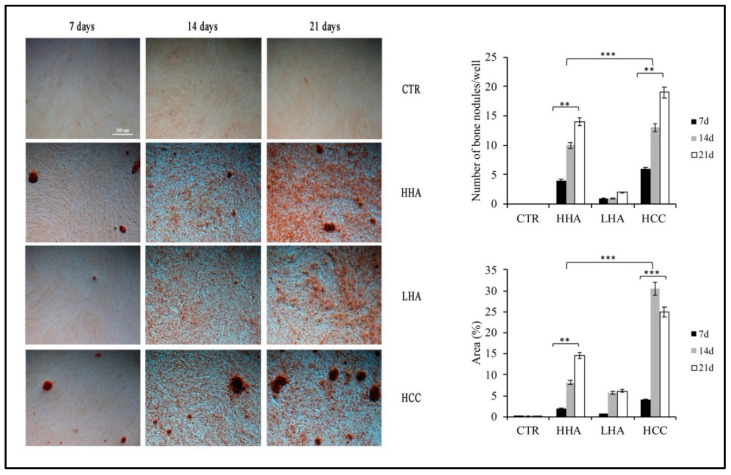
Calcification nodules evaluation at 7,14 and 21 days by Alizarin Red S in standard medium. Already at 7 days, Alizarin Red staining showed the formation of calcification nodules in all treated samples. At 14 and 21 days, HHA and HCC led to a greater number of calcification nuclei compared to LHA and control. This was confirmed also by calculating the percentage area of positive staining. The results are expressed as the mean ± SD of three independent experiments. ** *p* < 0.01 HHA vs. CTR, *** *p* < 0.001 HCC vs. CTR; *** *p* < 0.001 HHA vs. HCC.

We found that, in the standard medium all hyaluronans promoted the formation of calcification nodules already at 7 days. On the contrary, at 14 and 21 days, we observed that HCC and HHA led to a greater number of nodules compared to LHA and control (Figure 2).

This was confirmed also by calculating the area percentage of positive staining (Figure 2). HHA and HCC induced a significant increase in the percentage of the positively stained surface compared to those of LHA and control (*p* < 0.01 HHA vs. control, *p* < 0.001 HCC vs. control). In the osteogenic medium, the behavior was similar. Here, at 7 days, calcification nodules were already detectable (Figure S2).

At 14 and 21 days, HCC and HHA promoted a significant formation of calcification nodules (*p* < 0.01 HHA and HCC vs. control) associated with an increase of the percentage of positively stained surface compared to those of LHA and control (*p* < 0.01 HHA and HCC vs. control).

These data further confirmed that HCC, and then HHA, promoted the bone differentiation of hDPSCs in standard medium.

### 3.4. Hyaluronan-Based Gels Promoted Expression of CD44 in hDPSCs

**Figure 3:**
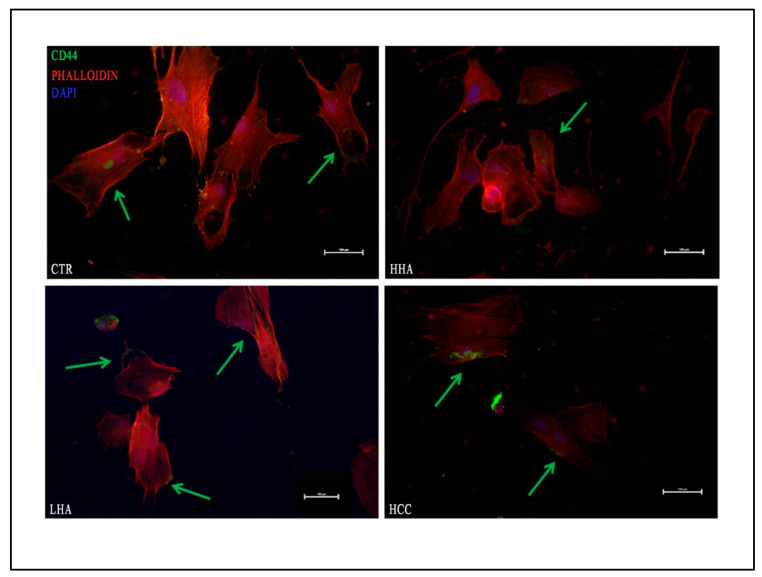
Evaluation of CD44 expression in hDPSCs after hyaluronans treatment in standard medium. CD44 marker was mainly localized on the hDPSCs cell surface with more areas of positivity after HCC treatment. CD44 in green, phalloidin in red; nuclei in blue. Green arrow indicates CD44 positivity. Scale bar: 100 μm.

Because one of the main receptors of the HA is the transmembrane glycoprotein CD44, we evaluated its expression in the different treatments and conditions by immunofluorescence. As reported in Figure 3, CD44 marker was mainly distributed on the hDPSCs cell surface.

In particular, in standard medium (Figure 3), we found that CD44 was highly expressed in all hyaluronans treatments and HCC showed specific areas of positivity on hDPSCs surface, while, in osteogenic medium, CD44 was distributed as puncta (Figure S3).

### 3.5. Hyaluronan-Based Gels Positively Regulated Gene Expression of YAP/TAZ, CTGF, ANKDR-1 and RUNX-1 and RUNX-2 in hDPSCs

YAP/TAZ are known to have a key function in stem cell differentiation [20,21] but no data are available on how YAP and TAZ are regulated by Hyaluronans. In our models, using qRT-PCR analyses, we found that *YAP* and *TAZ* are differently expressed.

**Figure 4:**
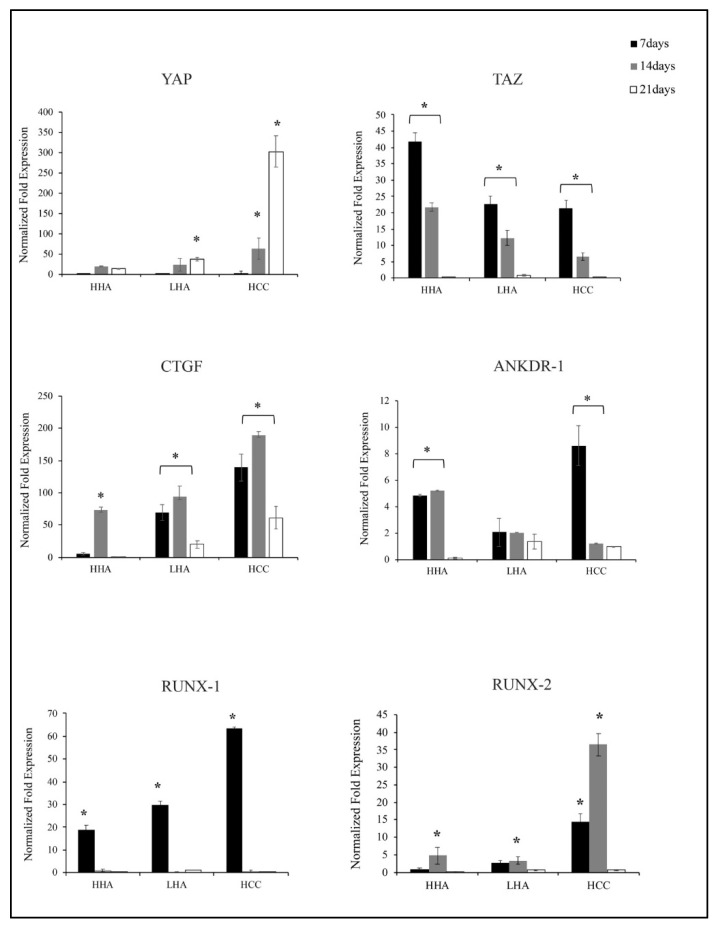
Analysis of *YAP*, *TAZ*, *CTGF*, *ANKDR-1*, *RUNX-1* and *RUNX-2* gene expression at 7, 14 and 21 days by qRT-PCR in standard medium. *YAP* is slightly upregulated from 14 to 21 days by HHA, LHA, and strongly activated by HCC; *TAZ* expression increased earlier, at 7 and 14 days, in particular with HHA; *CTGF* mRNA levels were upregulated by all HA-based gels up to 14 days, with a better upregulation exerted by HCC; *ANKDR-1* gene expression was strongly upregulated by HCC already at 7 days; *RUNX-1* gene expression was increased by all hyaluronans at 7 days, with a better effect of HCC; *RUNX-2* increased with HCC up to 14 days. The results are expressed as the mean ± SD of three independent experiments. * *p* < 0.01 vs. CTR.

Specifically, in standard medium, *YAP* is slightly upregulated from 14 to 21 days by HHA, LHA, and strongly activated by HCC (*p* < 0.01) (Figure 4).

On the contrary, *TAZ* followed a different expression kinetic. Indeed, *TAZ* expression increased earlier, at 7 and 14 days, in particular with HHA. In the presence of LHA or HCC, *TAZ* mRNA levels were less upregulated vs. control than it was in the presence of HHA (*p* < 0.01). As expected, *TAZ* is inversely modulated than *YAP*. Additionally, we next studied the expression of the well characterized YAP/TAZ target genes *CTGF* and *ANKDR1* [22]. We found that all hyaluronans increased *CTGF* and *ANKDR-1* gene levels. CTGF mRNA levels were upregulated by all HA-based gels up to 14 days, with a better upregulation exerted by HCC. For *ANKDR-1* gene expression, the upregulation was found for HHA at 7 and 14 days. HCC strongly increased *ANKDR-1* expression (*p* < 0.01) already at 7 days (Figure 4).

Then, we examined mRNA levels of *RUNX-1* and *2*, target genes of the YAP/TAZ pathway [22,23].

In the standard medium, all hyaluronans upregulated *RUNX-1* expression at 7 days, with a better effect exerted by HCC. *RUNX-2* was positively modulated by HCC up to 14 days (Figure 4).

### 3.6. Inhibition of YAP/TAZ Pathway Is Associated with a Reduction of Osteogenic Differentiation Exercised by Hyaluronans

To confirm that hyaluronans promote osteogenesis by activating the YAP/TAZ pathway, we treated hDPSCs with the YAP/TAZ inhibitor-1. Since this inhibitor is able to stop cell growth, we started by determining the noncytotoxic dose for hDPSCs. For this purpose, hDPSCs were exposed to different concentrations of YAP/TAZ inhibitor-1 (50, 25, 12.5, 7.25, 5, 2.5, 1.25 and 0.75 nM) for 24, 48, 72 h and 7 days. We did not detect appreciable differences in cell growth up to 72 h of treatment and up to a concentration of 2.5 nM (Figure S4), which we consequently chose as work concentration.

Next, we treated the hDPSCs with hyaluronans and YAP/TAZ inhibitor-1 in standard medium and evaluated both the expression of bone markers at 24, 72 h, 7 days and nuclear translocation of YAP/TAZ at 72 h.

**Figure 5:**
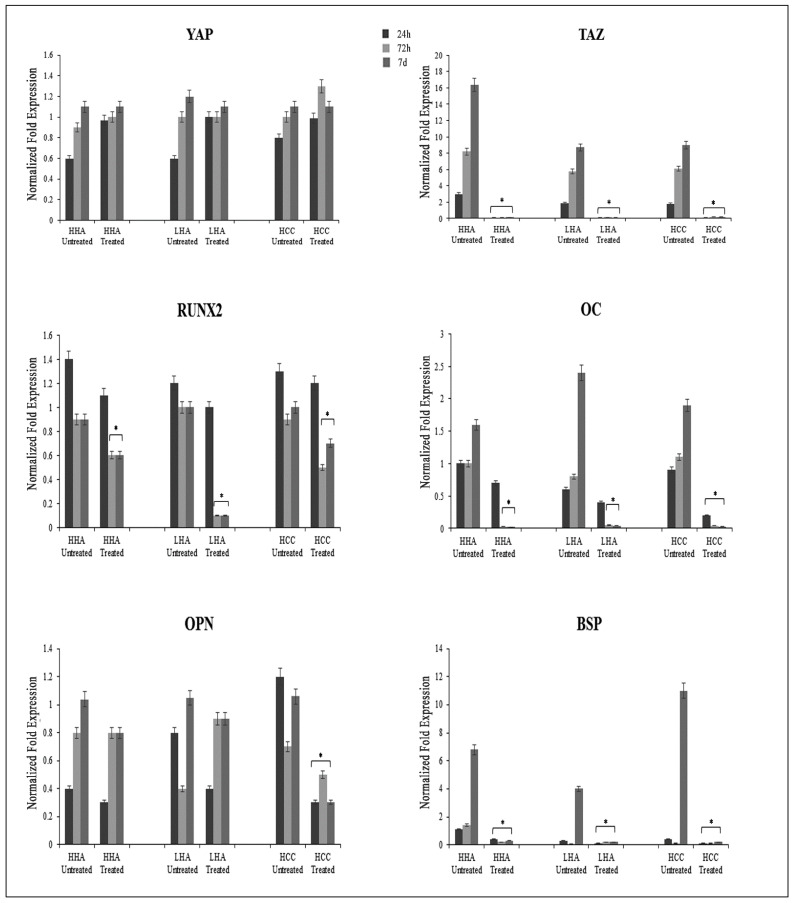
Analysis of *YAP*, *TAZ*, *RUNX-2*, *OC*, *OPN* and *BSP* gene expression at 24, 72 h and 7 days by qRT-PCR in standard medium after YAP/TAZ inhibitor-1 treatment. The expression of *YAP* slightly increases after YAP/TAZ inhibitor-1 treatment; *TAZ* expression is drastically reduced in treated cells; *RUNX-2* mRNA levels are strongly downregulated in treated cells, in particular at 72 h and 7 d; all osteogenic related markers, *OC*, *OPN* and *BSP*, are strongly reduced in treated cells. The results are expressed as the mean ± SD of three independent experiments. * *p* < 0.01 vs. Untreated.

After YAP/TAZ inhibitor-1 treatment, we observed a slight increase of *YAP* mRNA levels, and a drastic reduction of *TAZ* mRNA levels independently by times and hyaluronans treatment respect to untreated cells. Then, we assessed how TAZ depletion affected osteogenic differentiation. We found that all osteogenic related genes, including *RUNX2*, *OC*, *OPN* and *BSP* were strongly downregulated. In particular, we found that inhibitor effect was stronger in those cells treated with HCC rather than those treated with HHA and LHA for *OC*, and *BSP* at 72 h and 7 days (Figure 5).

**Figure 6:**
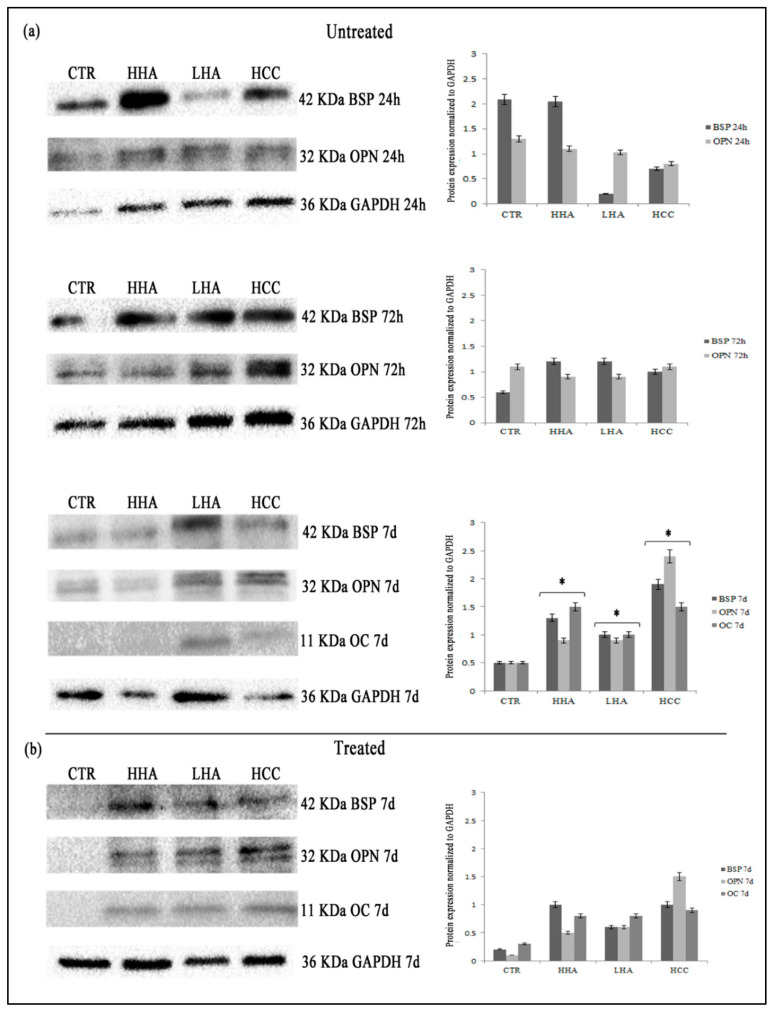
Analysis of OC, OPN and BSP protein expression at 24, 72 h and 7 days by Western blotting in standard medium after YAP/TAZ inhibitor-1 treatment. The expression of OC, OPN and BSP is completely depleted in cells treated with YAP/TAZ inhibitor-1 at 24 and 72 h (**a**). At 7 days only weak signal of protein expression is appreciable (**b**). The results are expressed as the mean ± SD of three independent experiments. * *p* < 0.01 vs. Untreated.

Then, we also examined the protein expression of bone related markers, by western blotting. In untreated cells, OC expression was detectable only at 7 days, whereas BSP and OPN were expressed already at 24 h. In YAP/TAZ inhibitor-1 treated cells, we observed that at 24 and 72 h, OC, OPN and BSP expression was absent. These markers were detectable only at 7 days and their expression was reduced compared to untreated hDPSCs (Figure 6).

**Figure 7:**
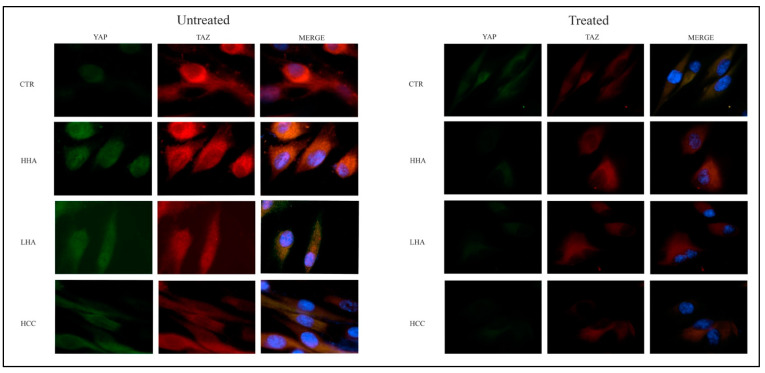
Analysis of YAP and TAZ, expression at 72 h by immunofluorescence in standard medium after YAP/TAZ inhibitor-1 treatment. The expression of YAP and TAZ was clearly evident both at cytoplasmic and nuclear level in untreated cells; after YAP/TAZ inhibitor-1 treatment, YAP and TAZ expression was strongly reduced, and the localization was predominantly in cytoplasm. Original magnification: 100×.

The activation of YAP/TAZ pathway led to the translocation of YAP/TAZ from cytoplasm in the nucleus. TAZ binds to YAP in cytoplasm and then the complex translocates in the nucleus. In our models, we observed by immunofluorescence that in untreated hDPSCs, the expression of YAP and TAZ was clearly evident both at cytoplasmic and nuclear level, indicating that YAP/TAZ pathway is activated. On the contrary, after YAP/TAZ inhibitor-1 treatment, YAP and TAZ expression was strongly reduced, and the localization was predominantly in the cytoplasm (Figure 7).

Taken together, these results show that hyaluronans promoted the bone differentiation via YAP/TAZ pathway.

## 4. Discussion

HA is a biopolymer largely used in clinical applications and regenerative medicine for its distinctive characteristics such as biocompatibility, biodegradability, and non-immunogenicity [24]. Positive effects of HA are reported in several conditions, yet there is a widespread fog regarding the HA’s molecular weights effects on different biological processes. This applies especially to osteogenic differentiation and osteoblast activity where the effects of HA are neglected. In this study, we show that HA formulations based on different molecular weights have different impact on hDPSCs osteogenic differentiation. We also show that HA induces YAP/TAZ signaling to promote osteogenic genes and matrix formation.

hDPSCs have been extensively used as a model for osteogenesis as they are naturally committed to osteogenic phenotype [10,11]. They have been used to test the osteoconductivity of different biomaterials such as titanium implants, as well as the capacity of drugs to induce osteogenesis both in vitro and in vivo [25]. Moreover, they were successfully grafted in humans to repair bone defects [26]. The osteogenic differentiation is a multistep process characterized by three main stages: proliferation, matrix production, and mineralization. It is regulated by different genes including *OC*, *BSP*, and *OPN* that are temporally and spatially controlled. Osteocalcin is the most abundant protein of bone where it plays a key role in the differentiation of osteoblast progenitors, with significant upregulation during both matrix synthesis and mineralization. BSP promotes osteoblast differentiation and functions as a calcification nucleator in the early phases of mineralization [27,28,29,30,31]. Osteopontin is considered as a late effector of mineralization [32]. The three hyaluronans formulations (HCC, LHA, HHA) increased OC, BSP, and OPN expression promoting osteogenic differentiation, with HCC being the most effective. HCC stimulates an upregulation of bone related markers already after 7 days of treatment, and matrix formation at 7–14 days as demonstrated by Alizarin Red S staining. This is of paramount interest for clinical use of HA, as these results were obtained in standard medium, without any osteogenic inducer. HCC, but also HHA, may be used to stimulate and promote the bone differentiation of endogenous stem cells improving the regeneration of damaged tissue.

HA is the main ligand of CD44 [33]; thus, we have then evaluated CD44 expression by hDPSCs after hyaluronans treatment. We found that HCC promoted CD44 expression in specific areas of hDPSCs membrane, suggesting that it binds to CD44 with greater efficiency than other formulations for which the CD44 distribution was not homogeneous. Previous studies have showed that CD44 is an upstream regulator of YAP/TAZ signaling [34]. We found that HCC induced a strong upregulation of *YAP*, *RUNX-1* and *RUNX-2* and downregulated *TAZ* already at 7 days of treatment. Moreover, HCC also promoted the gene expression of *CTGF* and *ANKDR1*, YAP/TAZ downstream target genes [22]. This indicated that HCC increases YAP/TAZ transcriptional activity, which promotes bone differentiation. The other hyaluronans are also capable of enhancing the activity of YAP/TAZ, but to a lesser extent than HCC.

To confirm the role of hyaluronans in the activation of YAP/TAZ pathway, we inhibited this pathway using YAP/TAZ inhibitor-1. After treatment, *TAZ* expression was completely depleted and this was correlated with a strong downregulation of all osteogenic markers, both at gene and protein levels. We found that TAZ depletion inhibited the formation of YAP/TAZ complex in the cytoplasm and its translocation into the nucleus. Following this, downstream genes, including bone related genes, were not activated. Taking into consideration all the above, we suggest that HCC binds CD44 on hDPSCs surface and induces the activation of YAP/TAZ pathway, with YAP and TAZ translocation into the nucleus and expression of bone related markers promoting bone differentiation.

## 5. Conclusions

Taken together, our data demonstrated that HCC induces and accelerates osteogenic differentiation and improves the mineralization via YAP/TAZ pathway. HCC shows greater stability than other linear HA formulations and a wide array of bioactive effects. HCC has the potential to stimulate the stem cells to differentiate in bone tissue without any bone inducing factors. This gel is easy to inject due to low viscosity; notwithstanding a high HA concentration in respect to other commercialized formulations. It may act in vivo as a viscous and adhesive gel to precisely stimulate the endogenous stem cells to differentiate in the injured site, supporting the translational value of the presented results toward new clinical approaches aiming at bone regeneration, especially in small bone defects.

## Figures and Tables

**Table 1:** Oligonucleotide sequences used for quantitative PCR.

Gene	Name	Sequence	Ta
*OC*	Osteocalcin	Forward CTCCACATCCTCGCCCTATTG Reverse CTTGGACACAAAGGCTGCAC	58 °C
*OPN*	Osteopontin	Forward GCCGAGGTGATAAGTGTGGTT Reverse TGAGGTGATGTCCTCGTTCTG	58 °C
*BSP*	Bone sialo protein	Forward CTGGCACAGGGTATACAGGGTAG Reverse ACTGGTGCCGTTTATGCCTTG	60 °C
*YAP*	Yes-associated protein 1	Forward CAACTCCAACCAGCAGCAAC Reverse TTGGTAACTGGCTACGCAGG	55 °C
*TAZ*	Transcriptional co-activator with PDZ-binding motif	Forward TGGACCAAGTACATGAACCACC Reverse TGCCTTCTATGCTCCCTCCT	55 °C
*hCTGF*	human-Connective tissue growth factor	Forward AGGAGTGGGTGTGTGACGA Reverse CCAGGCAGTTGGCTCTAATC	57 °C
*hANKDR1*	human-Ankyrin repeat domain-containing protein 1	Forward AGTAGAGGAACTGGTCACTGG Reverse TGGGCTAGAAGTGTCTTCAGAT	57 °C
*RUNX-1*	Runt domain transcription factors 1	Forward AACCCAGCATAGTGGTCAGC Reverse CATGGCTGCGGTAGCATTTC	57 °C
*RUNX-2*	Runt domain transcription factors 2	Forward ACCAGCAGCACTCCATATCTCTAC Reverse CTTCCATCAGCGTCAACACCATC	57 °C
*HRPT*	Hypoxanthine-guanine phosphoribosyltransferase	Forward TGACCTTGATTTATTTTGCATACC Reverse CGAGCAAGACGTTCAGTCCT	60 °C

## Data Availability

All data generated or analyzed during this study are included in this published article (and its supplementary information files).

## Supplementary Materials

The following are available online at https://www.mdpi.com/article/10.3390/cells10112899/s1, Figure S1: Analysis of OC, OPN and BSP protein expression at 7, 14 and 21 days by qRT-PCR and western blotting both in osteogenic medium, Figure S2: Calcification nodules evaluation at 7, 14 and 21 days by Alizarin Red S in osteogenic medium. Calcification nodules were detectable already at 7 days, Figure S3: Evaluation of CD44 expression in hDPSCs after hyaluronans treatment in osteogenic medium, Figure S4: Evaluation of hDPSCs viability after YAP/TAZ inhibitor-1 treatment.
