# Supplementary material for: Hyaluronan-Based Gel Promotes Human Dental Pulp Stem Cells Bone Differentiation by Activating YAP/TAZ Pathway
Source: Cells. 2021 Oct 26;10(11):2899. doi: 10.3390/cells10112899 (PMC8616223; doi:10.3390/cells10112899)
Supplement: Supplementary file 1 [file cells-10-02899-s001.zip › cells-1405491-supplementary.pdf]

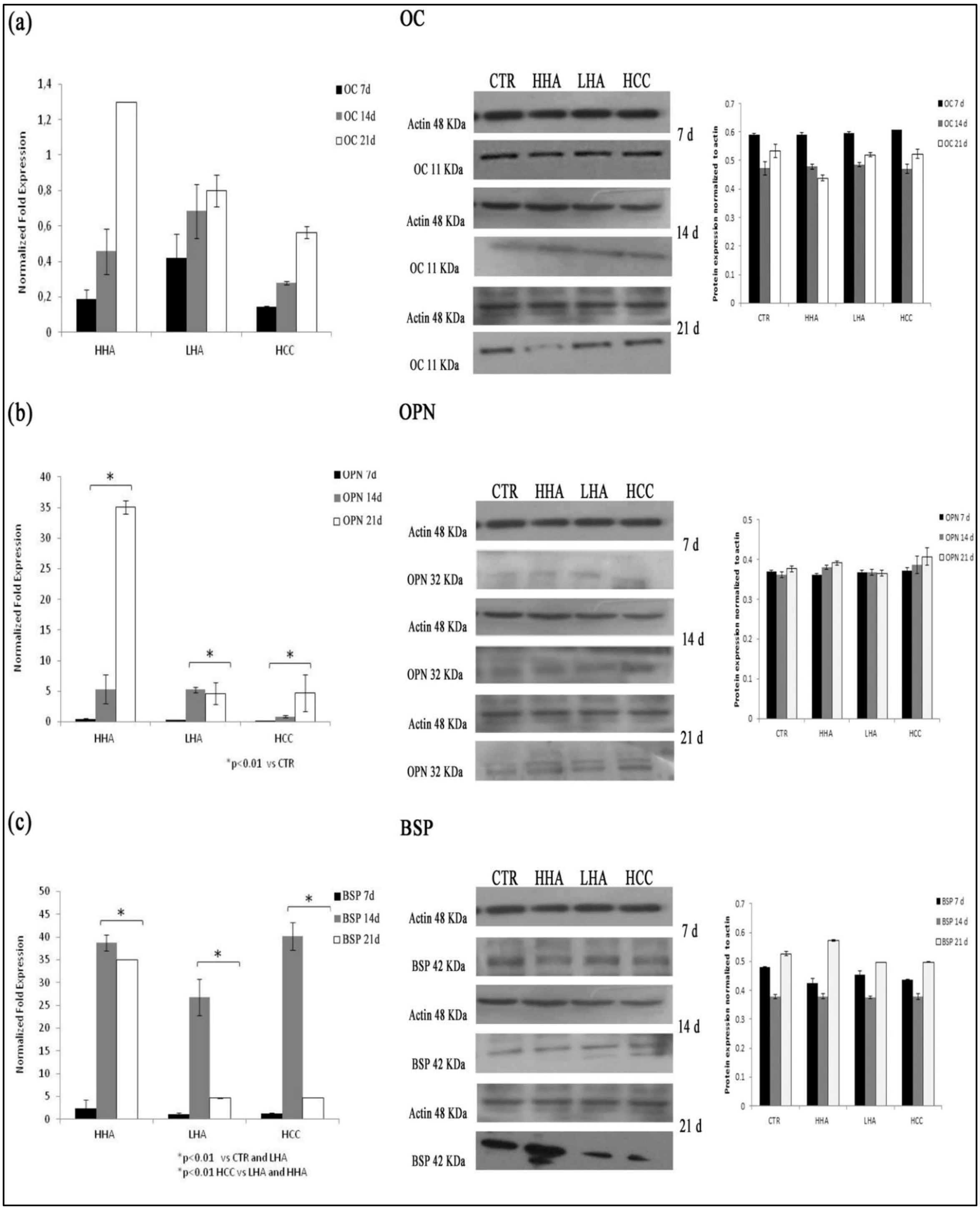


**Figure S1.** Analysis of OC, OPN and BSP protein expression at 7, 14 and 21 days by qRT-PCR and western blotting both in osteogenic medium. (**a**) OC gene expression was slightly upregulated in cells treated with hyaluronans; no significant differences were appreciable at protein level; (**b**) OPN gene expression strongly increased after HHA treatment at 21 days; no significant differences were appreciable at protein level; (**c**) BSP gene expression increased strongly at 14 days after hyaluronans treatment; no significant differences were appreciable at protein level. The results are expressed as the mean ±SD of three independent experiments. * $p < 0.01$ versus CTR and other hyaluronans; * $p < 0.05$ versus CTR.

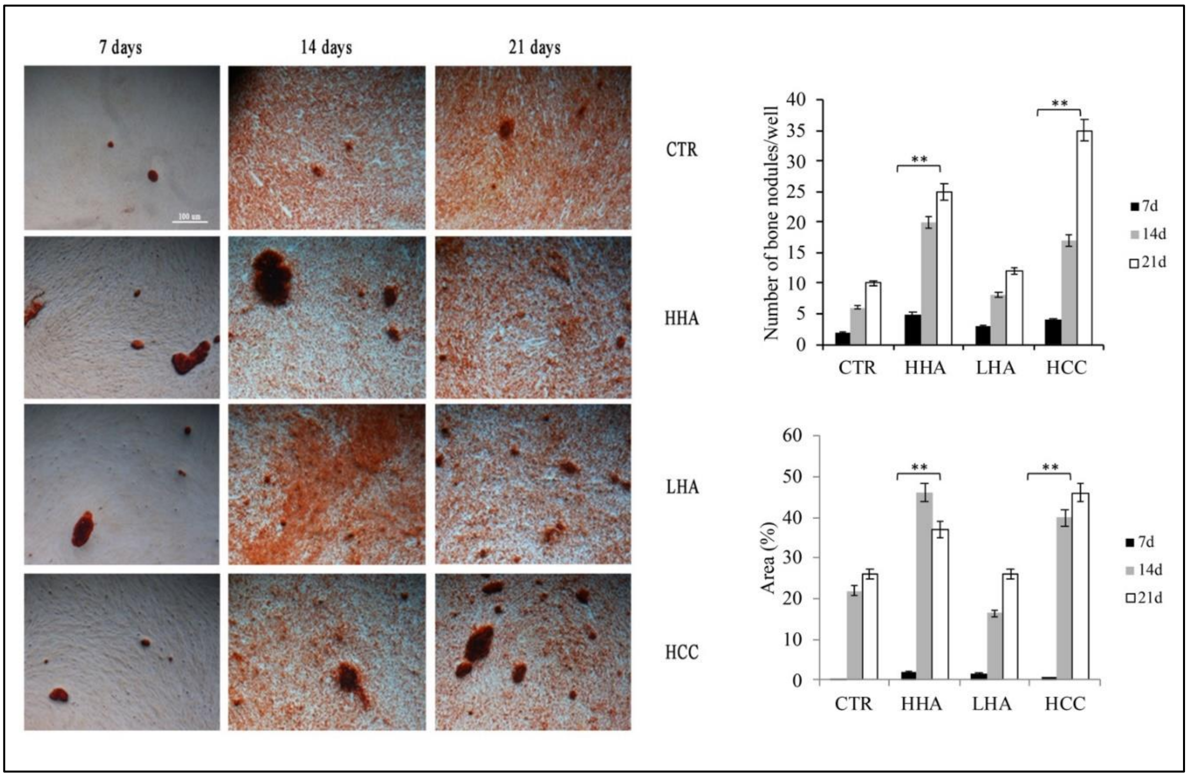


**Figure S2.** Calcification nodules evaluation at 7, 14 and 21 days by Alizarin Red S in osteogenic medium. Calcification nodules were detectable already at 7 days. At 14 and 21 days, HCC and HHA promoted a significant formation of calcification nodules associated with an increase of the percentage of positively stained surface respect to those of LHA and control. The results are expressed as the mean ±SD of three independent experiments. ** $p < 0.01$ HHA and HCC vs. CTR.

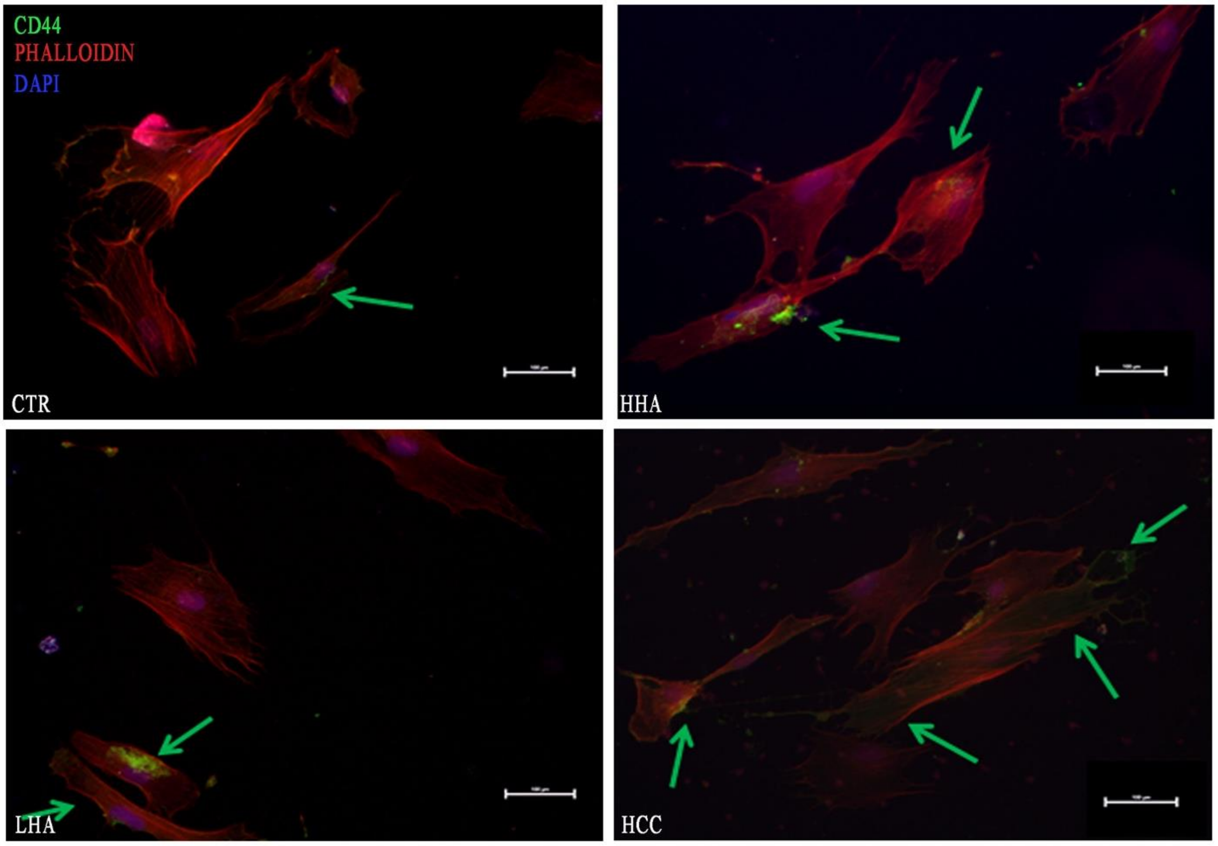


**Figure S3.** Evaluation of CD44 expression in hDPSCs after hyaluronans treatment in osteogenic medium. CD44 expression in osteogenic medium appeared as puncta. CD44 in green, phalloidin in red; nuclei in blue. Scale bar: 100 μm.

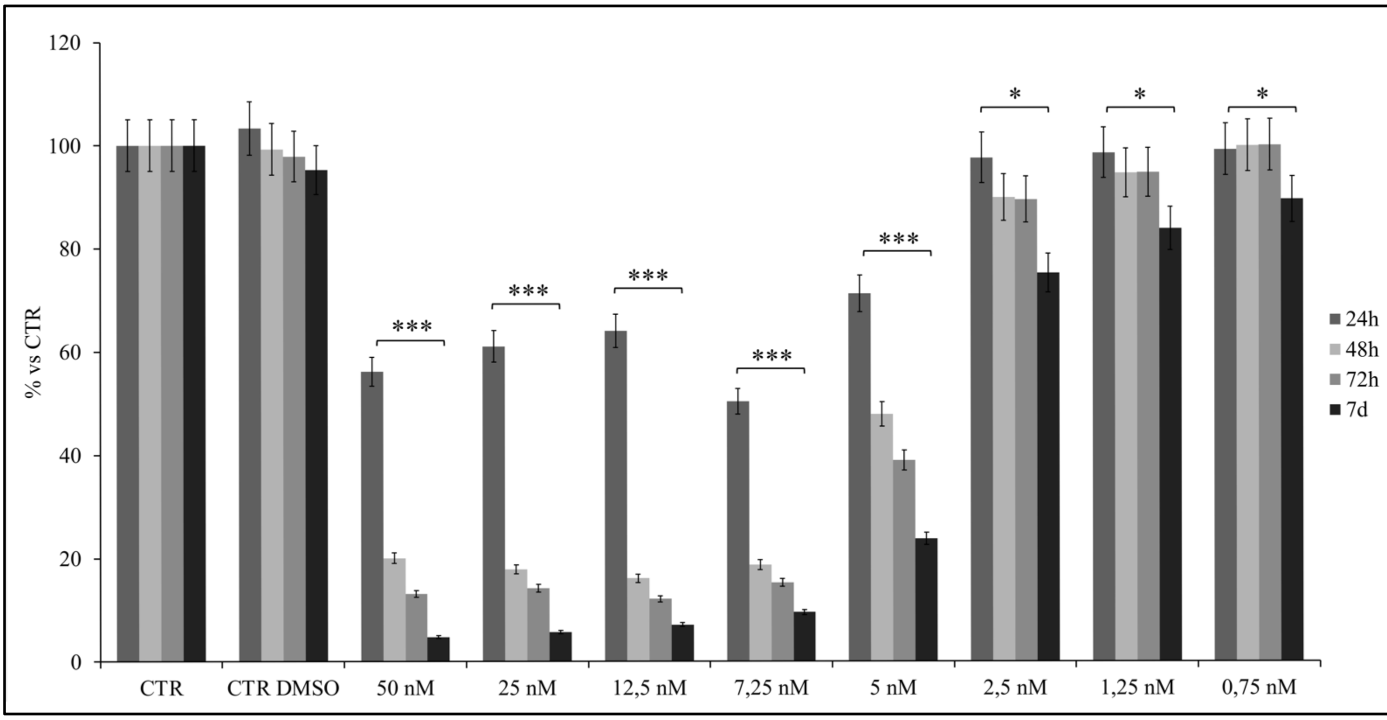


**Figure S4.** Evaluation of hDPSCs viability after YAP/TAZ inhibitor-1 treatment. No differences in cell growth up to 72 h of treatment and up to a concentration of 2.5 nM were appreciable. At this concentration the decrease in cell viability at 7 days is normally due to the overgrowth of cells for long times. Concentrations between 50 and 5 nM were extremely toxic. * $p < 0.01$ vs. CTR; *** $p < 0.001$ vs. CTR.
